# Food Neophobias in Spanish Adults with Overweight or Obesity by Sex: Their Association with Sociodemographic Factors and the Most Prevalent Chronic Diseases

**DOI:** 10.3390/foods13132030

**Published:** 2024-06-26

**Authors:** Carmen del Campo, Cristina Bouzas, Margalida Monserrat-Mesquida, Josep A. Tur

**Affiliations:** 1 Research Group on Community Nutrition & Oxidative Stress, University of the Balearic Islands-IUNICS, 07122 Palma de Mallorca, Spainmargalida.monserrat@uib.es (M.M.-M.); 2 CIBEROBN (Physiopathology of Obesity and Nutrition), Instituto de Salud Carlos III, 28029 Madrid, Spain; 3 Health Research Institute of Balearic Islands (IdISBa), 07120 Palma, Spain

**Keywords:** food neophobias, food habits, high prevalent diseases, adults, Spain

## Abstract

Food neophobia has been defined as the reluctance to try new foods. Food neophobia is common in children and older people, but until now, scarce research has been carried out on food neophobia in the adult population. The aim of this study was to assess the most usual food neophobias in Spanish adults with overweight and obesity by sex, and their association with sociodemographic factors and the most prevalent chronic diseases. A cross-sectional observational study was carried out on adults (mean age of 43.5 ± 13.7 years old; *n* = 590; 50% female) with overweight or obesity. Their anthropometrics, adherence to the Mediterranean diet, age, educational level, economic level, smoking and sleeping habits, physical activity, chronic diseases, and food neophobias were assessed. The highest food neophobias in Spanish adults with overweight or obesity were directed toward vegetables, meat, fish, pulses, game meat, and fruits, mostly among females, with differences between sexes. Phobias of the soft texture of foods were also observed, without differences between sexes. Age, educational level, sleeping habits, and physical activity were directly related, and economical level and smoking were inversely related to food neophobia, mainly to healthy foods, and more obvious in males than in females. There were associations between body mass index (BMI) and chronic diseases and food neophobia. Adherence to the adaption of healthy and sustainable diets is low within food neophobics, increasing the risk of diet-related chronic diseases.

## 1. Introduction

Food neophobia is defined as the reluctance to try new or unfamiliar foods and measured by means of the degree of acceptance or rejection that individuals feel for foods that are not part of their usual diet [1], which has been well researched in children but not in adults [2]. This dislike for one or more foods could be considered an adaptive reaction of human beings to protect themselves from contamination or disease and could be also considered an emotion related to food [3]. Consequently, disgust motivates avoidance behavior and triggers specific disgust stimuli. Tactile and disgust sensitivity have been reported as the main sensory and emotional variables associated with food neophobia, creating dislike of a food based on negative memories or experiences of tasting new foods [2]. The response of disgust is characterized by parasympathetic activation, activation of specific facial muscles, appraisals of contamination, and oral rejection. This unwillingness to try new foods, which, in many cases, extends to familiar foods, is usually linked to strong food preferences [4].

It has been pointed out that different levels of food neophobia depend on the geographical area [5,6], as well as sociodemographic variables, such as sex [7], age [1,5,7,8,9], educational level [7], and employment level [10]. It was also pointed out that the impact of food neophobia emerges independently, regardless of weight, age, socioeconomic status, gender, or lifestyle [11]. However, there is evidence that the heritability of neophobia is very present in its development [12], representing more than 70% of the risk of developing it. The importance of personality and temperament, both in children and adults, should be also considered [3].

Food neophobics tend to present restrictive consumption behaviors of certain foods, which may be considered a risk factor for eating disorders [13]. A 7-year-follow up of Finnish and Estonian cohorts showed that food neophobia was closely linked to adverse eating patterns and reduced dietary variety and quality [14], and it was inversely associated with food group variety and fruit and vegetable variety [15]. Most studies have pointed out that neophobia could be linked to low body mass index (BMI) scores [16,17], with some exceptions showing higher BMI scores [18,19]. It was also reported that Spanish children with food neophobia consumed less fruits, vegetables, cereals [14], and fish, have higher consumption of sweets, and have unbalanced breakfasts [20], affecting their diet quality. It was described that adults with obesity showed greater preferences for high-fat foods and rejected vegetables [21]. So, food neophobics showed low intakes of fiber, proteins, and monounsaturated fatty acids, and high intakes of saturated fat and salt, which are associated with an increased risk of obesity and noncommunicable diseases, including cardiovascular disease and type 2 diabetes mellitus [11]. There is evidence that the development of obesity in adults is related to taste acuity, since population a study showed that people with obesity had a higher taste threshold and a lower number of fungiform papillae than adults with normal weight, which is related to more pronounced taste for products with high energy density (related to fatty taste stimuli) [22]. Food neophobia is common in children and older people [23], but until now, scarce research has been carried out on food neophobia in the adult population [11]. A study showed that fussy or neophobic behaviors in childhood pose a higher risk of neophobic behaviors in adulthood [24].

Neophobia can have an important variable and subjective components, depending on the environmental factors to which an individual is exposed. Except in the case of food intolerances and allergies, the reason for rejection can be measured based on the symptoms caused, according to clinical guidelines [25,26]. Food allergies and intolerances and celiac disease are the main disorders that cause the rejection of certain foods because they cause moderate to severe clinical symptoms.

The aim of this study was to assess the most usual food neophobias in Spanish adults with overweight and obesity by sex, and their association with sociodemographic factors and the most prevalent chronic diseases.

## 2. Methods

### 2.1. Design and Sample Recruitment

A cross-sectional observational study was carried out on adults (mean age of 43.5 ± 13.7 years old; *n* = 590; 50% female) with overweight or obesity, according to age and sex-specific WHO reference values [27], who were recruited in the Castilla La Mancha region, Spain, in February 2022. The exclusion criteria were patients with diagnosed food allergies, an immature or diminished mental capacity to be able to communicate their food neophobias, physical injuries that prevented communication, pregnancy, prosthesis and/or edema, and weight loss higher than 6 kg in the last five years. This study was approved by the Medicinal Research Ethics Committee of the Ciudad Real General University Hospital, Spain (ref. C-498), and the written consent of the participants was obtained.

### 2.2. Anthropometrics and Sociodemographic Data

Anthropometric measurements were made according to standard procedures and sex, with the participants dressed in light clothing and barefoot. Height was measured to the nearest millimeter, with the participant’s head maintained in the Frankfurt horizontal plane, using a mobile stadiometer (Seca 213, SECA Deutschland, Hamburg, Germany). Body weight (kg) was measured with a bioimpedance device (Inbody 120, Microcaya, Bilbao, Spain). Body mass index (BMI) was calculated as body weight (kg) divided by the square of body height (m^2^). BMI standard deviation scores were calculated using age and sex-specific WHO reference values [27], as normal weight (BMI = 18.5 to ≤25 kg/m^2^), overweight (BMI = 25 to ≤30 kg/m^2^), and obesity (BMI ≥30 kg/m^2^). Waist circumference was measured twice using an anthropometric tape (Seca 201, SECA Deutschland, Hamburg, Germany) in a standing position, midway between the last rib and the iliac crest. The mean of the two measurements was recorded to the nearest millimeter [28]. Waist-to-hip ratio (WHR) cut-off points (≥0.90 for men; ≥0.85 for women) were used to define abdominal obesity or excessive accumulation of fat at the abdomen [29].

Age, educational level (primary/no studies, secondary, or university), economic level (unemployment, self-employed, or employee), smoking (yes or no), and sleeping habits (≥8 h/d or <8 h/d), physical activity (none/light or moderate/vigorous), and chronic diseases (hypothyroidism, hypertension, type 2 diabetes mellitus, and hypercholesterolemia), were also registered using questionnaires. The levels of physical activity were classified according to the WHO recommendations for adults aged 18–64 years, defining two levels: participants who did moderate/vigorous physical activity (at least 75–150 minutes/day of vigorous-intensity aerobic physical activity, or 150–300 minutes/day of moderate-intensity aerobic physical activity, or an equivalent combination of moderate- and vigorous-intensity activity throughout the week); and participants who did not, classified as light or no physical activity [30].

### 2.3. Adherence to the Mediterranean Diet (MedDiet)

Adherence to the MedDiet [31] was assessed by means of a previously used, validated 14-item MedDiet adherence questionnaire [32]. A score was given for each met objective: 1 (compliance) or 0 (non-compliance). The total score ranged between 0 and 14, with a score of 0 indicating no compliance, and a score of 14 indicating maximum adherence.

### 2.4. Food Neophobia Assessment

The Food Neophobia Scale (FNS) [33] was developed and validated in Canadian psychology students and has been widely used since 1992. Over the years, many countries have become ethnically and culturally diverse with worldwide globalization [34]; thus, certain FNS questions may not reflect food neophobia nowadays, especially those related to ethnic meals. Although it has been recently updated by removing two items due to ambiguity and cultural inappropriateness [35], in this current study, food neophobia was assessed by means of more simplistic questions that avoided multicultural and ethnic questions.

Food neophobias in adults were assessed by orally asking the participants if they had a reluctance to try new food types (game meat, meat, fish, vegetables, fruits, pulses, milk and dairy products, grains, sugar and sweets, nuts, coffee, and eggs), or to soft, hard, or boiled textures. In the interview, the participants were asked to answer ‘yes’ or ‘no’ regarding the foods with the highest level of phobia, as well as if this avoidance was related to the texture. The score shows the percentages of avoided or rejected foods.

### 2.5. Statistics

SPSS statistical software package version 29.0 (SPPS Inc., Chicago, IL, USA) was used for the analysis. The data are shown as the mean and standard deviation (SD), except for the prevalence data, which are expressed as percentages. Differences in the categorical variables between the groups were analyzed through a Chi-squared test. For the sociodemographic continuous variables, their distribution was analyzed. The variables had a normal distribution. Hence, Student’s *t*-test was used to assess differences between the sexes. The logistic regression, odds ratio, and 95% confidence interval (OR (95% CI)) were fitted to evaluate each one of the items of food neophobia (as dependent variables) and their association with adherence to the MedDiet, sociodemographic factors, and chronic diseases (as independent variables), adjusted by age, educational level, economical level, sleeping habits, smoking habits, physical activity, and BMI.

## 3. Results

The characteristics of the respondents by sex are shown in Table 1. Males showed higher body weight, BMI, socioeconomic level, educational level, physical activity level, and hypertension than females. Females showed higher abdominal obesity than males.

Table 2 shows the food neophobias in the studied adult population by sex. Males showed higher phobias of game meat, grains, and coffee than females, and females showed higher phobias of vegetables and meat than males. Since there were few phobias of sugar and sweets, nuts, coffee, and egg consumption, these foods were not considered in the following analyses. Since the highest phobia was to soft textures, just this was considered in the following analyses.

Table 3 shows the associations between food neophobia and quality of diet, expressed as adherence to the Mediterranean diet (MedDiet) by sex. Phobias of meat and milk and dairy consumption are associated with a high adherence to the MedDiet in women, whereas phobias of fish and fruit consumption in both males and females are linked to a lower adherence to the MedDiet.

Table 4 and Table 5 show the associations between food neophobias and sociodemographic factors in male and female adults, respectively. In males (Table 4), inverse associations were observed between age and phobias of game meat, vegetable, and pulse consumption, with direct associations between age and phobias of fish and grain consumption. Inverse associations between educational level and phobias of game meat, vegetable, meat, fish, and fruit consumption were observed. There were direct associations between economical level and phobias of game meat and meat consumption, and inverse associations were found between economic level and phobias of fruit and grain consumption and textures; sleeping habits and phobias of grain consumption; and smoking habits and phobias of fish consumption and textures. Inverse associations between physical activity and phobias of game meat, pulse, and grain consumption and textures were found, with direct associations between physical activity and phobias of vegetable, meat, and fruit consumption. Direct associations between BMI and phobias of game meat, meat, and pulse consumption were found, with an inverse association between BMI and a phobia of fish consumption. In females (Table 5), age was inversely associated with phobias of game meat, vegetable, fish, pulse, fruit, and milk and dairy product consumption, and a direct associated between age and a phobia of grain consumption was found. Economical level was associated with a phobia of fish consumption and inversely associated with a phobia of fruit consumption. Smoking was associated with a phobia of game meat consumption, and inversely associated with phobias of fish, pulse, and fruit consumption.

Table 6 and Table 7 show the associations between food neophobia and chronic diseases in the male and female participants, respectively. In males (Table 6), hypothyroidism was associated with phobias of game meat, vegetable, and grain consumption, and inversely associated with a phobia of fish consumption; hypertension was associated with phobias of meat and grain consumption, and inversely associated with a phobias of fruit consumption and textures; T2DM was inversely associated with phobias of vegetable, meat, pulse, and fruit consumption; hypercholesterolemia was inversely associated with phobias of game meat, milk and dairy product, and grain consumption and textures. In females (Table 7), hyperthyroidism was associated with a phobia of meat consumption, and inversely associated with a phobia of pulse consumption; hypertension was inversely associated with a phobias of pulse consumption and textures; T2DM was inversely associated with phobias of fish and fruit consumption; hypercholesterolemia was associated with a phobia of pulse consumption.

## 4. Discussion

The main findings of the current study were that most food neophobias in Spanish adults with overweight or obesity were directed toward vegetables, meat, fish, pulses, game meat, and fruits, mostly among females, with differences between sexes. Phobias of soft-textured foods were also observed, without differences between the sexes. The quality of diet in the current study, expressed as adherence to MedDiet, was associated with phobias of the consumption of meat and milk and dairy products, and inversely associated with phobias of fish and fruits, which are related to the MedDiet concept [20,31].

Although few studies have assessed the effects of food neophobia in adults, evidence linking food neophobia to detrimental dietary intakes in adults is emerging [15]. As with children, high food neophobia has been associated with low dietary variety, low intake of several food groups, and low diet quality [15,36,37]. Previous studies have reported that adults with high food neophobia tend to have low consumption rates of nutrient-dense foods like vegetables and fish [3,13,15,37,38,39]. Other studies have found high food neophobia is inversely related with highly recommended foods, like fruits, vegetables, fish, and whole-grain bread [13,15,37,40]. Italian adults showed an inverse association between high food neophobia and adherence to the Mediterranean diet [40]. Another study showed that food neophobia is linked to low dietary quality in adults [11]. Despite these findings, Portuguese adults showed no significant relationships between diet quality or nutrient intake and food neophobia [39], and Polish food neophobics showed high consumption of meat and meat products, suggested to be due to sociocultural conditions [41]. This last determinant could also be applied to the inverse association between dietary quality and the phobia of meat found in the Spanish adults in this study, since their region shows one of the highest meat consumptions in Spain [34], and familiarity would be a prominent motivator of food choices for these individuals [42].

Therefore, previous results and the current findings both suggest that the effects of food neophobia on achieving dietary recommendations are not very pronounced. It preferably decreases the intake of beneficial food, and it may be different across countries. Thus, the assessment of the influence of food neophobia on a population in a country or region demands specific research, justifying the current study on the assessment of food neophobia among Spanish adults and that food neophobia is related to dietary quality throughout one’s lifespan, not only in children.

The current findings show that age, educational level, sleeping habits, and physical activity are directly related, and economical level and smoking habits are inversely related to food neophobia, especially toward healthy foods. The males and females in this study showed similar relationships; however, the association between food neophobia and vegetable intake as well as other nutrient-dense foods was more obvious in males than in females, as previously reported [9,18,38,39,43]. However, some authors have found that women are more neophobic than men [44,45], while others have reported the contrary [14,38]. It has been found that food neophobics are older [9,41], have a lower level of education [7,46], and have a higher BMI; the latter relationship has been more observed in women than in men [18,38]. A high prevalence of food neophobia was previously detected in people with low socioeconomic status [18,47]. A poor economic situation has been usually associated with a high level of food neophobia [9,10,39,41,48], and the current results agree with those previous findings. Therefore, the current results agree with previous studies reporting that food neophobia is associated with age, sex, education level, socioeconomic status, and living region [7,9,15,49,50].

The current findings also show an association between BMI and phobias of game meat, meat, and grain consumption, and an inverse association with fish consumption in males but not in females, which agree with previous studies reporting a relationships between high food neophobia and overweight and obesity [3,7,15,39,41,49,50] and between lower overall diet quality and BMI [3,50].

Phobia of game meat, vegetables, and milk and dairy products were associated with hyperthyroidism; phobias of meat and grains were associated with hypertension; phobias of game meat, milk and dairy products, grains, and soft textures were inversely associated with hypercholesterolemia. T2DM was associated with phobias of vegetables, meat, pulses, and fruits in males, and with phobias of fish and fruit consumption in females. The relationship between high food neophobia and an increased risk of T2DM was previously shown [34]. Perhaps diabetics mistakenly believe that foods considered healthy (vegetables, pulses, fruits, etc.) have more sugars than other foods, and therefore, they exclude them from their diet, which is in line with the accepted concept that food neophobia is a protective mechanism against the intake of potentially harmful foods [3].

In this sense, excessive sugar consumption, and more recently, the increase in high fructose syrup, has attracted attention due to its negative effect on people’s health, especially in promoting weight gain and increasing diabetes and cardiovascular diseases [51]. Perhaps for this reason, diabetic participants showed a greater phobia of consuming fruits. However, a small intake of fructose contained in fruits of a low glycemic index appeared to reduce concentrations of postprandial glucose and improved glycated hemoglobin (HbA1c) and high-density lipoprotein–cholesterol (HDL-chol) levels [51]. Epidemiological evidence shows that populations with high fish consumption have a lower risk of T2DM [51]. However, Castilla La Mancha, the Spanish region where the participants were recruited, shows one of the lowest consumption of fish in Spain, lower than the Spanish mean consumption [52]. Therefore, there was not a health-related relationship between fish consumption and diabetes mellitus among the Castilla La Mancha inhabitants, but this is rather a result of the characteristic eating habits in this region.

The exclusion of healthy foods may cause the opposite effect, since a low-quality diet increases the risk of chronic diseases, such as T2DM [34], and low dietary quality was associated with an increased risk of subsequent chronic diseases (e.g., obesity, cardiovascular disease, coronary heart disease, T2DM, and inflammation) [11,53,54,55,56]. Therefore, adherence to the adaption of healthy and sustainable diets is low among food neophobics, increasing their risk of diet-related chronic diseases [40]. Further research applying prospective intervention educational programs in diabetics is needed to solve nutritional mistakes.

All these results show that food phobias and preferences acquired during childhood may persist into adulthood, as previously suggested [18,57]. The dietary habits of adults are consequence of long-term eating habits, which begin in youth and develop throughout life, tending to be poorly diverse diets with a narrow range of meal choices, and leading to malnutrition and an increased risk of frailty [58]. Food neophobia has been found to be more prevalent in older people, especially for protein-rich foods [11,40,58], and mostly in females, agreeing with the current results, which is especially important in older adults as a factor risk of future diseases [11,28,58].

However, food neophobia is not an unchangeable personality trait. It has been pointed out that educational programs that increase familiarity and exposure to foods, creating positive experiences, are able to reduce food neophobia, and hence, increase the quality of diet and decrease the prevalence of related chronic diseases [42].

## 5. Strengths and Limitations of the Study

This study contributes to the emerging knowledge on food neophobia among adults and its link to chronic diseases. However, the current study also has several limitations. The Food Neophobia Scale (FNS) was developed in 1992 and developed and validated in Canadian psychology students [33]. Since then, it has been widely used, but over the years, many countries have become ethnically and culturally diverse with worldwide globalization [34], so certain FNS questions may not reflect food neophobia nowadays, especially those related to ethnic meals. Although it has been recently updated by removing two items due to ambiguity and cultural inappropriateness [35], in the current study, food neophobia was assessed by means of more simplistic questions that avoided multicultural and ethnic questions. The current findings are also specific to the Spanish cultural background and should be used with caution in relation to other populations; further research is necessary to confirm the observed relationships under different sociocultural and regional conditions. The cross-sectional design does not allow for assessing the causality of relationships among outcomes, and further prospective studies are needed.

## 6. Conclusions

The most common food neophobias in Spanish adults with overweight or obesity were directed toward vegetables, meat, fish, pulses, game meat, and fruits, mostly among females, with differences between the sexes. Age, educational level, sleeping habits, and physical activity were directly related, and economical level and smoking habit were inversely related to food neophobia, especially to healthy foods, and more obviously in males than in females. There were associations between BMI and chronic diseases and food neophobia. Adherence to the adaption of healthy and sustainable diets is low among food neophobics, increasing the risk of diet-related chronic diseases.

## Figures and Tables

**Table 1:** Characteristics of the respondents.

	Total (*n* = 590)	Males (*n* = 294)	Females (*n* = 296)	*p*-Value
Age (yr)	43.8 ± 13.7	41.8 ± 13.0	45.2 ± 14.2	0.049 *
Body weight (kg)	88.9 ± 18.5	97.1 ± 17.6	80.9 ± 15.5	<0.001 *
BMI (kg/m^2^)	31.8 ± 5.9	32.3 ± 5.7	31.4 ± 6.1	0.015 *
Waist-to-hip ratio	0.97 ± 0.07	0.97 ± 0.06	0.97 ± 0.08	0.547 *
Abdominal obesity	64.9%	29.8%	99.0%	<0.001 ^#^
Educational level				0.002 ^#^
Primary/no studies	61.5%	58.5%	64.5%	
Secondary	31.6%	36.2%	27.2%	
University	6.8%	5.3%	8.3%	
Economical level				<0.001 ^#^
Unemployment	23.3%	8.5%	37.6%	
Self-employed	23.8%	35.1%	12.7%	
Employee	53.0%	56.4%	49.7%	
Sleeping habits				0.181 ^#^
≥8 h/d	47.9%	45.9%	49.8%	
<8 h/d	52.1%	54.1%	50.2%	
Smoking habit	76.2%	76.5%	75.9%	0.814 ^#^
Physical activity				<0.001 ^#^
None/light	79.7%	71.4%	87.4%	
Moderate/vigorous	20.3%	28.6%	12.0%	
Chronic diseases				
Hypothyroidism	14.9%	16.0%	13.8%	0.304 ^#^
Hypertension	31.8%	38.3%	25.5%	<0.001 ^#^
T2DM	8.2%	9.6%	6.9%	0.099 ^#^
Hypercholesterolemia	21.2%	21.3%	21.0%	0.920 ^#^
Adherence to MedDiet	6.7 ± 1.7	6.7 ± 1.6	6.6 ± 1.8	0.256 *

Abbreviations: BMI: body mass index. MedDiet: Mediterranean diet; T2DM: type 2 diabetes mellitus. * Mean values ± standard deviation and differences in males vs. females by Student’s *t*-test; ^#^ percentages and differences in males vs. females by ꭓ^2^.

**Table 2:** Food neophobias in the studied adult population by sex.

Food Neophobia	Males (*n* = 294)	Females (*n* = 296)	*p*-Value *
Phobia of game meat	20.4%	29.6%	<0.001
Phobia of vegetables	58.6%	64.8%	0.031
Phobia of meat	42.4%	49.7%	0.002
Phobia of fish	29.8%	31.0%	0.647
Phobia of pulses	24.5%	26.6%	0.419
Phobia of fruits	12.8%	14.5%	0.404
Phobia of milk and dairy products	7.4%	3.8%	0.126
Phobia of grains	7.4%	4.4%	0.007
Phobia of sugar and sweets	1.1%	1.4%	0.628
Phobia of nuts	1.1%	0.3%	0.145
Phobia of coffee	1.1%	0.0%	0.012
Phobia of eggs	1.1%	1.0%	0.961
Phobia of textures	24.5%	22.7%	0.486
Soft texture	18.8%	16.3%	0.621
Hard texture	0.0%	0.6%	-
Boiled texture	5.9%	4.8%	-

* Males vs. females by ꭓ^2^.

**Table 3:** Association (OR; 95%CI) between food neophobia and quality of diet, expressed as adherence to MedDiet) by sex.

Food Neophobia	Adherence to MedDiet in Males (*n* = 294)	Adherence to MedDiet in Females (*n* = 296)
Phobia of game meat	0.62 (0.36–1.07)	0.91 (0.60–1.36)
*p*-value	0.086	0.636
Phobia of vegetables	0.71 (0.46–1.10)	1.20 (0.81–1.78)
*p*-value	0.127	0.372
Phobia of meat	1.44 (0.93–2.22)	1.89 (1.27–2.83)
*p*-value	0.101	**0.002**
Phobia of fish	0.43 (0.27–0.67)	0.85 (0.73–1.27)
*p*-value	**<0.001**	0.781
Phobia of pulses	0.67 (0.40–1.09)	0.79 (0.52–1.21)
*p*-value	0.108	0.280
Phobia of fruits	0.21 (0.08–0.53)	0.35 (0.21–0.57)
*p*-value	**0.001**	**0.001**
Phobia of milk and dairy products	1.13 (0.47–2.75)	2.30 (1.31–4.04)
*p*-value	0.782	**0.004**
Phobia of grains	3.10 (0.95–5.10)	1.52 (0.58–3.94)
*p*-value	0.060	0.392
Phobia of textures (soft)	0.91 (0.84–1.51)	1.23 (0.80–1.88)
*p*-value	0.707	0.351

Abbreviations: CI: confidence interval; MedDiet: Mediterranean diet; OR: odds ratio. OR reference (1.00): no neophobia. OR analysis was adjusted by age, educational level, economical level, sleeping habits, smoking habit, physical activity, and BMI.

**Table 4:** Associations between food neophobias and sociodemographic factors in adult male participants.

Food Neophobia	Age	Educational Level	Economical Level	Sleeping Habits	Smoking Habit	Physical Activity	BMI
Game meat							
OR (95%CI)	0.41 (0.24–0.69)	0.42 (0.29–0.63)	2.32 (1.39–3.87)	1.14 (0.71–1.81)	1.57 (0.91–2.71)	0.23 (0.12–0.45)	1.67 (1.05–3.66)
*p*-value	**<0.001**	**<0.001**	**0.001**	0.584	0.103	**<0.001**	**0.031**
Vegetables							
OR (95%CI)	0.30 (0.20–0.46)	0.54 (0.36–0.83)	0.87 (0.67–1.13)	0.88 (0.63–1.23)	1.36 (0.91–2.02)	1.75 (1.20–2.56)	0.90 (0.75–1.07)
*p*-value	**<0.001**	**0.005**	0.311	0.459	0.135	**0.004**	0.233
Meat							
OR (95%CI)	1.08 (0.72–1.62)	0.57 (0.41–0.80)	1.81 (1.22–2.70)	0.85 (0.56–1.27)	2.85 (1.73–4.69)	1.66 (1.06–2.60)	1.94 (1.25–3.04)
*p*-value	0.702	**<0.001**	**0.003**	0.420	**<0.001**	**0.026**	**0.003**
Fish							
OR (95%CI)	2.14 (1.36–3.35)	0.49 (0.34–0.71)	0.97 (0.64–1.47)	0.99 (0.66–1.49)	0.35 (0.21–0.59)	1.13 (0.72–1.78)	0.36 (0.22–0.59)
*p*-value	**<0.001**	**<0.001**	0.870	0.960	**<0.001**	0.599	**<0.001**
Pulses							
OR (95%CI)	0.55 (0.35–0.87)	0.67 (0.44–1.04)	1.00 (0.74–1.34)	0.83 (0.54–1.28)	1.13 (0.69–1.86)	0.58 (0.30–0.82)	1.62 (0.98–2.69)
*p*-value	**0.011**	0.072	0.992	0.403	0.633	**0.007**	0.060
Fruits							
OR (95%CI)	0.96 (0.49–1.90)	0.25 (0.15–0.44)	0.41 (0.23–0.76)	0.61 (0.54–1.72)	1.76 (0.83–3.73)	6.14 (3.08–12.23)	0.86 (0.44–1.67)
*p*-value	0.914	**<0.001**	**0.004**	0.897	0.144	**<0.001**	0.652
Milk and dairy products							
OR (95%CI)	1.05 (0.47–3.37)	0.98 (0.49–1.98)	0.65 (0.32–1.31)	0.96 (0.63–2.64)	0.62 (0.19–1.99)	1.13 (0.0.47–2.73)	1.14 (0.58–1.39)
*p*-value	0.901	0.961	0.227	0.491	0.424	0.791	0.158
Grains							
OR (95%CI)	3.40 (1.57–5.41)	0.39 (0.13–1.20)	0.25 (0.16–0.39)	0.10 (0.03–0.41)	0.92 (0.73–1.17)	0.19 (0.50–0.69)	3.69 (1.88–5.10)
*p*-value	**<0.001**	0.100	**<0.001**	**0.001**	0.241	**0.012**	**0.001**
Textures (soft)							
OR (95%CI)	1.45 (0.89–2.34)	1.73 (1.11–2.69)	0.56 (0.35–0.89)	0.72 (0.46–1.13)	0.33 (0.20–0.55)	0.55 (0.32–0.932)	0.99 (0.59–1.68)
*p*-value	0.133	**0.015**	**0.013**	0.152	**<0.001**	**0.026**	0.987

Abbreviations: BMI: body mass index; CI: confidence interval; OR: odds ratio. OR reference (1.00): no neophobia. OR analysis was adjusted by age, educational level, economical level, sleeping habits, smoking habit, physical activity, and BMI.

**Table 5:** Associations between food neophobias and sociodemographic factors in adult female participants.

Food Neophobia	Age	Educational Level	Economical Level	Sleeping Habits	Smoking Habit	Physical Activity	BMI
Game meat							
OR (95%CI)	0.41 (0.27–0.62)	0.98 (0.72–1.32)	0.82 (0.47–1.43)	0.96 (0.66–1.39)	1.69 (1.08–2.67)	0.94 (0.52–1.68)	0.98 (0.560–1.60)
*p*-value	**<0.001**	0.877	0.488	0823	**0.023**	0.830	0927
Vegetables							
OR (95%CI)	0.45 (0.30–0.66)	1.01 (0.76–1.34)	0.66 (0.37–1.18)	1.16 (0.81–1.65)	0.79 (0.52–1.22)	0.99(0.57–1.71)	0.93 (0.58–1.49)
*p*-value	**<0.001**	0.974	0.164	0.415	0.292	0.969	0.76
Meat							
OR (95%CI)	1.03 (0.71–1.48)	0.86 (0.66–1.13)	0.78 (0.46–1.32)	0.80 (0.57–1.12)	1.22 (0.82–1.80)	1.10 (0.66–1.868)	1.48 (0.95–2.32)
*p*-value	0.891	0.291	0.351	0.200	0.329	0.707	0.085
Fish							
OR (95%CI)	0.64 (0.43–0.96)	0.99 (0.74–1.34)	2.61 (1.32–5.15)	1.20 (0.83–1.72)	0.64 (0.43–0.98)	0.97 (0.55–1.70)	0.94 (0.58–1.53)
*p*-value	** 0.031 **	0.985	**0.006**	0.333	**0.038**	0.906	0.799
Pulses							
OR (95%CI)	0.98 (0.96–0.99)	0.95 (0.69–1.31)	0.99 (0.54–1.81)	1.19 (0.82–1.73)	0.61 (0.40–0.92)	0.80 (0.44–1.44)	0.96 (0.59–1.57)
*p*-value	**<0.001**	0.763	0.984	0.347	**0.018**	0.456	0.887
Fruits							
OR (95%CI)	0.53 (0.35–0.81)	1.01 (0.39–1.50)	0.37 (0.19–0.71)	1.36 (0.92–2.00)	0.58 (0.38–0.91)	0.92 (0.49–1.70)	0.75 (0.39–1.44)
*p*-value	**0.004**	0.958	**0.003**	0.116	**0.016**	0.784	0.382
Milk and dairy products							
OR (95%CI)	0.40 (021–0.75)	0.92 (0.39–2.20)	0.89 (0.67–1.20)	0.93 (0.53–1.65)	1.74 (0.93–4.02)	1.15 (0.50–2.66)	0.53 (0.23–1.22)
*p*-value	**0.004**	0.856	0.456	0.816	0.077	0.728	0.136
Grains							
OR (95%CI)	4.20 (1.33–7.20)	0.85 (0.41–1.76)	1.55 (0.23–1.44)	0.57 (0.23–1.35)	0.82 (0.30–2.25)	0.82 (0.63–1.27)	1.16 (0.56–4.69)
*p*-value	**0.014**	0.658	0.581	0.238	0.700	0.341	0.379
Textures (soft)							
OR (95%CI)	0.72 (0.46–1.11)	0.84 (0.61–1.15)	0.57 (0.32–1.02)	1.48 (0.98–2.21)	1.04 (0.65–1.67)	1.17 (0.64–2.13)	0.79 (0.45–1.39)
*p*-value	0.138	0.286	0.056	0.059	0.868	0.610	0.412

Abbreviations: BMI: body mass index; CI: confidence interval; OR: odds ratio. OR reference (1.00): no neophobia. OR analysis was adjusted by age, educational level, economical level, sleeping habits, smoking habit, physical activity, and BMI.

**Table 6:** Associations between food neophobia and chronic diseases in male adult participants.

Food Neophobia	Hypothyroidism	Hypertension	T2DM	Hypercholesterolemia
Game meat				
OR (95%CI)	2.15 (1.03–4.67)	1.01 (0.63–1.62)	0.48 (0.21–1.10)	0.43 (0.25–0.75)
*p*-value	**0.050**	0.969	0.082	**0.003**
Vegetables				
OR (95%CI)	2.97 (1.71–5.16)	0.77 (0.52–1.14)	0.35 (0.17–0.72)	0.69 (0.43–1.13)
*p*-value	**<0.001**	0.190	**0.004**	0.139
Meat				
OR (95%CI)	1.02 (0.60–1.74)	2.47 (1.66–3.67)	0.52 (0.28–0.98)	0.72 (0.45–1.13)
*p*-value	0.948	**<0.001**	**0.043**	0.155
Fish				
OR (95%CI)	0.46 (0.27–0.80)	1.36 (0.90–2.07)	0.86 (0.44–1.68)	0.61 (0.37–1.02)
*p*-value	**0.006**	0.145	0.664	0.061
Pulses				
OR (95%CI)	0.80 (0.42–1.50)	0.98 (0.64–1.51)	0.31 (0.16–0.60)	1.04 (0.61–1.78)
*p*-value	0.481	0.944	**<0.001**	0.886
Fruits				
OR (95%CI)	0.95 (0.46–1.98)	0.31 (0.17–0.57)	0.31 (0.13–0.73)	1.36 (0.61–3.04)
*p*-value	0.899	**<0.001**	**0.007**	0.457
Milk and dairy products				
OR (95%CI)	3.21 (1.04–9.88)	1.02 (0.48–2.20)	1.82 (0.17–3.95)	0.29 (0.11–0.73)
*p*-value	**0.042**	0.951	0.990	**0.009**
Grains				
OR (95%CI)	1.44 (0.44–4.68)	4.23 (1.30–7.79)	2.81 (0.10–4.85)	0.22 (0.73–0.66)
*p*-value	0.544	**0.017**	0.990	**0.007**
Textures				
OR (95%CI)	1.21 (0.63–2.33)	0.37 (0.23–0.58)	0.55 (0.27–1.13)	0.21 (0.12–0.36)
*p*-value	0.557	**<0.001**	0.104	**<0.001**

Abbreviations: CI: confidence interval; OR: odds ratio; T2DM: type 2 diabetes mellitus. OR reference (1.00): no neophobia. OR analysis was adjusted by age, educational level, economical level, sleeping habits, smoking habit, physical activity, and BMI.

**Table 7:** Associations between food neophobia and chronic diseases in female adult participants.

Food Neophobia	Hypothyroidism	Hypertension	T2DM	Hypercholesterolemia
Game meat				
OR (95%CI)	1.54 (0.85–2.80)	1.03 (0.67–1.58)	1.51 (0.73–3.27)	1.18 (0.74–1.86)
*p*-value	0.151	0.880	0.289	0.491
Vegetables				
OR (95%CI)	0.70 (0.35–1.39)	0.99 (0.66–1.48)	1.46 (0.74–2.88)	1.36 (0.89–2.08)
*p*-value	0.160	0.966	0.269	0.153
Meat				
OR (95%CI)	1.44 (0.87–2.40)	1.33 (0.91–1.95)	0.20 (0.07–0.57)	1.31 (087–1.98)
*p*-value	**0.022**	0.141	**0.003**	0.196
Fish				
OR (95%CI)	1.14 (0.65–2.00)	0.89 (0.59–1.35)	0.34 (0.14–0.83)	0.96 (0.61–1.51)
*p*-value	0.637	0.590	**0.017**	0.867
Pulses				
OR (95%CI)	0.43 (0.25–0.73)	1.70 (1.07–2.72)	1.27 (0.60–2.72)	1.99 (1.16–3.41)
*p*-value	**0.002**	**0.026**	0.533	**0.012**
Fruits				
OR (95%CI)	1.49 (0.70–3.16)	1.25 (0.70–2.25)	0.66 (0.28–1.52)	1.87 (0.95–3.67)
*p*-value	0.302	0.452	0.326	0.070
Milk and dairy products				
OR (95%CI)	1.29 (0.51–3.25)	0.59 (0.32–1.07)	0.99 (0.33–2.99)	0.69 (0.36–1.30)
*p*-value	0.588	0.083	0.984	0.248
Grains				
OR (95%CI)	2.67 (0.50–5.35)	0.95 (0.35–2.63)	0.91 (0.18–1.55)	1.15 (0.36–3.69)
*p*-value	0.253	0.926	0.910	0.805
Textures				
OR (95%CI)	1.31 (0.70–2.44)	0.63 (0.41–0.99)	1.37 (0.60–3.10)	0.91 (0.56–1.48)
*p*-value	0.396	**0.043**	0.454	0.706

Abbreviations: CI: confidence interval; OR: odds ratio; T2DM: type 2 diabetes mellitus. OR reference (1.00): no neophobia. OR analysis was adjusted by age, educational level, economical level, sleeping habits, smoking habit, physical activity, and BMI.

## Data Availability

There are restrictions on the availability of the data of this trial due to the signed consent agreements around data sharing, which only allow access to external researchers for studies following the project’s purposes. Requestors wishing to access the trial data used in this study can make a request by emailing pep.tur@uib.es.

## Abbreviations

BMI: body mass index; CI: confidence interval; FNS: Food Neophobia Scale; MedDiet: Mediterranean diet; HDL-chol: high-density lipoprotein–cholesterol; OR: odds ratio; SD: standard deviation; T2DM: type 2 diabetes mellitus; WHO: World Health organization; WHR: waist-to-hip ratio.
